# Effects of Transcranial Ultrasound Stimulation on Trigeminal Blink Reflex Excitability

**DOI:** 10.3390/brainsci11050645

**Published:** 2021-05-15

**Authors:** Andrea Guerra, Edoardo Vicenzini, Ettore Cioffi, Donato Colella, Antonio Cannavacciuolo, Silvia Pozzi, Barbara Caccia, Giulia Paparella, Giulia Di Stefano, Alfredo Berardelli, Matteo Bologna

**Affiliations:** 1 IRCCS Neuromed, 86077 Pozzilli, Italy; andrea.guerra@uniroma1.it (A.G.); giulia.paparella@uniroma1.it (G.P.); alfredo.berardelli@uniroma1.it (A.B.); 2 Department of Human Neurosciences, Sapienza University of Rome, 00185 Rome, Italy; edoardo.vicenzini@uniroma1.it (E.V.); ettore.cioffi@uniroma1.it (E.C.); donato.colella@uniroma1.it (D.C.); antonio.cannavacciuolo@uniroma1.it (A.C.); giulia.distefano@uniroma1.it (G.D.S.); 3 National Center for Radiation Protection and Computational Physics, Istituto Superiore di Sanità (ISS), 00161 Rome, Italy; silvia.pozzi@iss.it (S.P.); barbara.caccia@iss.it (B.C.)

**Keywords:** transcranial ultrasound stimulation (TUS), blink reflex, brainstem, excitability, GABA, interneurons, non-invasive brain stimulation, neuromodulation

## Abstract

Recent evidence indicates that transcranial ultrasound stimulation (TUS) modulates sensorimotor cortex excitability. However, no study has assessed possible TUS effects on the excitability of deeper brain areas, such as the brainstem. In this study, we investigated whether TUS delivered on the substantia nigra, superior colliculus, and nucleus raphe magnus modulates the excitability of trigeminal blink reflex, a reliable neurophysiological technique to assess brainstem functions in humans. The recovery cycle of the trigeminal blink reflex (interstimulus intervals of 250 and 500 ms) was tested before (T0), and 3 (T1) and 30 min (T2) after TUS. The effects of substantia nigra-TUS, superior colliculus-TUS, nucleus raphe magnus-TUS and sham-TUS were assessed in separate and randomized sessions. In the superior colliculus-TUS session, the conditioned R2 area increased at T1 compared with T0, while T2 and T0 values did not differ. Results were independent of the interstimulus intervals tested and were not related to trigeminal blink reflex baseline (T0) excitability. Conversely, the conditioned R2 area was comparable at T0, T1, and T2 in the nucleus raphe magnus-TUS and substantia nigra-TUS sessions. Our findings demonstrate that the excitability of brainstem circuits, as evaluated by testing the recovery cycle of the trigeminal blink reflex, can be increased by TUS. This result may reflect the modulation of inhibitory interneurons within the superior colliculus.

## 1. Introduction

Transcranial ultrasound stimulation (TUS) is a novel non-invasive brain stimulation technique that uses acoustic pressure waves delivered deep within tissues [1,2,3]. Ultrasound waves can be focused on a particular target with a specific spatial resolution based on wavelength and frequency. TUS can be transmitted through the skull and may exert direct modulation of neurons at low intensities [2,3,4]. The putative mechanism of TUS relates to the mechanical energy of ultrasound, which produces changes in cell membrane proteins and bioelectrical properties, including activation of voltage-dependent sodium and potassium, and calcium channels [5,6,7].

In humans, TUS of the primary motor cortex decreased corticospinal excitability and intracortical facilitation, increased GABAergic inhibition and reduced motor reaction task performance [8,9]. Conversely, motor evoked potential (MEP) amplitude was facilitated immediately after stimulation [10]. When delivered over the primary somatosensory cortex, TUS decreased somatosensory evoked potential amplitude [11], enhanced sensory discrimination task performance [11], and induced tactile sensations in contralateral hand regions [12,13]. Overall, these findings suggest that TUS can modulate sensorimotor functions in humans.

To date, whether TUS modifies the excitability of deeper brain structures in humans is unknown. Although one recent study showed somatosensory evoked potentials modulation by TUS on the thalamus [14], no study has assessed the possible effects of brainstem insonation. A reliable neurophysiological tool to investigate human brainstem circuits is the trigeminal blink reflex, elicited by electrical stimulation of the supraorbital nerve and mediated by large myelinated non-nociceptive fibers. The blink reflex is characterized by an early ipsilateral component (R1), which originates from an oligosynaptic pontine circuit, followed by a bilateral late component (R2), which reflects the activity of a polysynaptic circuit in the medullary reticular area [15,16,17,18]. Brainstem circuit excitability can be assessed through the R2 recovery cycle, a non-invasive neurophysiological technique based on paired stimulation at different interstimulus intervals (ISI) [15,19,20]. Various neural systems are involved in the control and modulation of the blink reflex, including descending pathways from cortical and subcortical areas [16,21,22,23,24,25,26].

In this study, we tested the effects of TUS on trigeminal blink reflex excitability, as assessed by the blink reflex recovery cycle, in healthy humans. For this purpose, we delivered TUS in different sessions targeting three brainstem areas, i.e., substantia nigra, superior colliculus, and nucleus raphe magnus. Due to the different roles of the three aforementioned nuclei on blink reflex circuit, e.g., the substantia nigra inhibits the superior colliculus which in turn excites the inhibitory activity of the nucleus raphe magnus [21,22], we hypothesize facilitatory or inhibitory effects of TUS on blink reflex excitability depending on the targeted area. A control session with sham stimulation was also performed. The blink reflex recovery cycle was assessed before and at two time-points after TUS, thus allowing us to investigate both the short- and long-term effects of TUS. A better understanding of the effects of TUS on blink reflex may guide novel non-invasive approaches for investigating and neuromodulating brainstem functions in healthy subjects and in pathological conditions [25].

## 2. Materials and Methods

### 2.1. Participants

Sixteen healthy subjects (7 females; mean age ± 1 SD: 27 ± 2.3 years; age range: 23–33 years) were consecutively enrolled at the Department of Human Neurosciences, Sapienza University of Rome. All subjects were right-handed as assessed by the Edinburgh Handedness Inventory (score ≥ 60) [27], and none presented a history of any neurological, psychiatric, eye, or eyelid disorder or were taking any drug potentially acting on the central nervous system. Other exclusion criteria were jet lag, irregular working hours, sleep restrictions in the last week or past drug abuse. All participants gave their informed consent to the experimental procedures, which were approved by the local ethics committee and conducted in accordance with the Declaration of Helsinki.

### 2.2. Transcranial Ultrasound Stimulation

**Figure 1:**
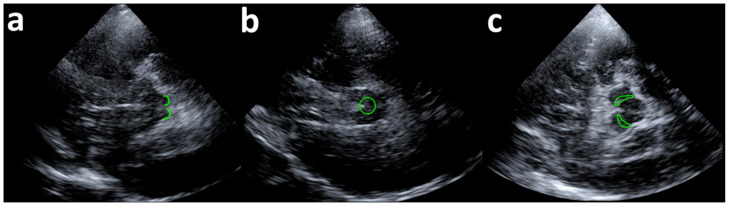
Ultrasound visualization of target brainstem nuclei. (**a**) Superior colliculus (SC); (**b**) Nucleus raphe magnus (NRM); (**c**) Substantia nigra (SN). The size of the targeted area was ~15 mm and brainstem nuclei were centred on the screen.

TUS was performed with a 4 P1 probe (Siemens S2000 apparatus, Siemens Medical, Solutions, WA, USA) with standard diagnostic transcranial imaging settings. B-Mode transtemporal right-sided imaging was obtained in the standard axial planes (midbrain, upper and lower pons, and cella media planes) [28] to identify the structures of interest. TUS insonation was performed for 180 s, with the mechanical index set to 100% (1.4; TIC 2.2), reducing the probe frequency to as low as possible (1.75 MHz) to maximize ultrasound beam penetration. Pulsed-wave Doppler (2 MHz, PRF 4340) was also switched on during insonation allowing the visualization of B-Mode imaging in real time simultaneously with pulse-wave Doppler. For each stimulation session, the following structures were identified, kept on screen, and insonated: the superior colliculus, nucleus raphe magnus, and substantia nigra [28] (Figure 1). Neuroanatomical landmarks were used to focus ultrasounds according to previous publications on the topic [29]. The sham stimulation was performed with the “freezing” machine ultrasound option.

**Figure 2:**
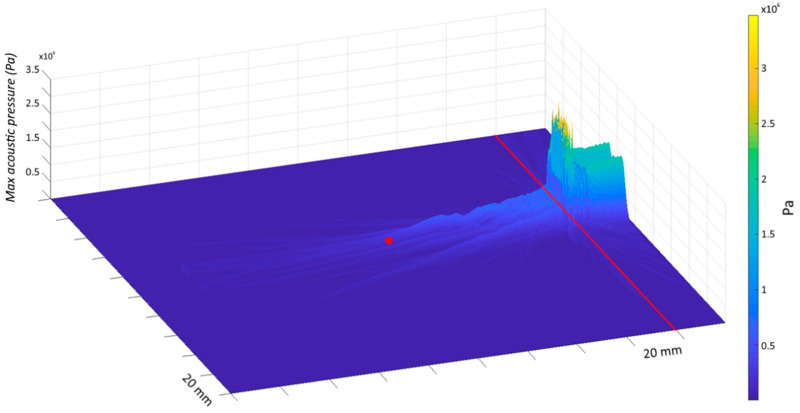
Ultrasound beam simulation. Pressure field of the ultrasound beam from the temporal bone (site of probe application—red line) to the target area in the brainstem (distance of ~80 mm—red dot). The coordinates of the target area have been determined from the brain scan atlas created by Neuromorphometrics, Inc. (https://scalablebrainatlas.incf.org/human/NMM1103, accessed on 5 February 2021), following the methods described in [31]. Based on our model, the pressure wave was reduced by ~75% from the temporal bone to the targeted site due to the skull attenuation (temporal bone thickness: 5 mm) and the estimated pressure of the ultrasound beam in the brainstem resulted 152 kPa.

An acoustic model was implemented to evaluate the attenuation of the ultrasound beam properties by the skull bone and to estimate the pressure value produced at the brainstem level. For the simulation, we used the characteristics of the transducer adopted in this study, a bone speed of sound of 3476 m/s and a midbrain speed of sound of 1546.3 m/s. The modelling was computed using the K-Wave toolbox in MATLAB [30] and the estimated pressure of the ultrasound beam in the brainstem resulted 152 kPa (Figure 2).

### 2.3. Blink Reflex and R2 Recovery Cycle

**Figure 3:**
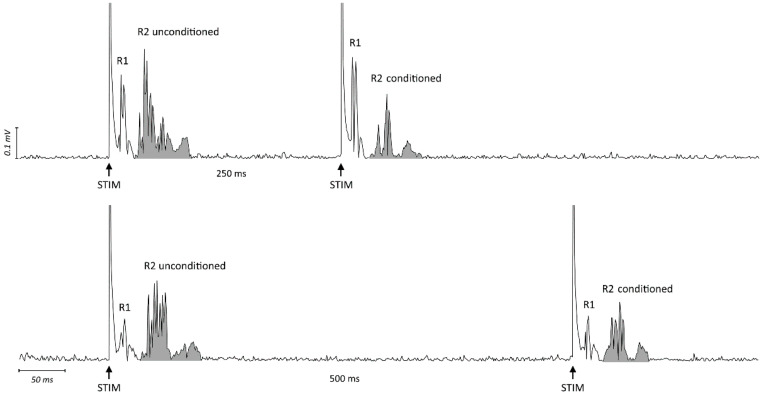
Raw EMG traces of blink reflex response to paired stimulation at ISI 250 ms (upper trace) and 500 ms (lower trace) in one representative subject. Shown are rectified traces recorded from the right orbicularis oculi muscle. The arrows indicate the timing of electric stimulation (STIM). The grey area represents the area under the curve (AUC) of the rectified EMG for the unconditioned and conditioned R2. The ratios between the AUC of conditioned R2 (paired stimulation) and unconditioned R2 (first stimulation) in the upper and lower trace were 0.44 and 0.67, respectively.

EMG recordings were obtained from the orbicularis oculi muscle bilaterally using surface electrodes. The cathode was placed over the muscle, the anode at the lateral angle of the eye. EMG signals were amplified using D360 amplifiers (Digitimer, Welwyn, UK), bandpass filtered (53–2500 Hz), analog-to-digital converted using a 1401 AD converter (CED, Cambridge, UK) at a sample rate of 5000 Hz, and collected on a computer. Electrical stimulation was applied to the right supraorbital nerve with a bipolar stimulating electrode and constant current generator (Digitimer). The supraorbital nerve was stimulated immediately above the supraorbital notch with the cathode positioned above the supraorbital foramen and the anode about 2 cm higher and laterally rotated. All stimuli had a 0.2 ms duration and stimulus intensity was set at approximately 3 times the R2 threshold (lowest intensity with an R2 of ≥ 50 µV response in at least 5 out of 10 trials). The blink reflex response to paired stimulation was assessed at ISI of 250 and 500 ms as this is a commonly applied methodology to test the inhibitory phase of the R2 recovery cycle [32,33,34]. Trials with background EMG ≥ 100 µV were rejected online. Data were analyzed offline using Signal software (Cambridge Electronic Design, UK). Raw blink recordings were DC-corrected, rectified, and averaged [35]. The onset and offset of the conditioned and unconditioned R2 responses were manually determined for each trial. The area under the curve of the rectified EMG was then measured and the ratio between conditioned and unconditioned R2 areas was calculated (Figure 3). Measures were analyzed offline by two investigators who were unaware of the session type.

### 2.4. Experimental Procedure

Participants were seated on a chair, at rest, with their eyes open in a quiet room with normal indoor lighting. All subjects underwent four different experimental sessions, conducted in a random order and on separate days: (1) superior colliculus-TUS; (2) nucleus raphe magnus-TUS; (3) substantia nigra-TUS; (4) sham-TUS. In each session, both participants and investigators were blind to the stimulation condition, except for the researcher who delivered TUS. The R2 recovery cycle was assessed by 10 paired stimuli (5 with 250 ms ISI and 5 with 500 ms ISI) applied in a random order, with a 30–45 s intertrial interval to avoid habituation. Blink reflex responses were recorded before (T0), 3 (T1—early effect), and 30 min (T2—late effect) after TUS.

### 2.5. Statistical Analysis

Data were analyzed using repeated measures analyses of variance (rmANOVAs). A first rmANOVA with ‘CONDITION’ (4 levels: superior colliculus-TUS, nucleus raphe magnus-TUS, substantia nigra-TUS, and sham-TUS), ‘SIDE’ (2 levels: right and left) and ‘TIME-POINT’ (3 levels: T0, T1, and T2) was used to compare the area of unconditioned R2 between the different sessions and time-points. Another rmANOVA with ‘CONDITION’, ‘SIDE’, ‘TIME-POINT’, and ‘ISI’ (2 levels: 250 ms and 500 ms) was applied to assess possible changes in the area of conditioned R2. Paired t-tests were also used to specifically compare the area of unconditioned R2 at T0 between sessions. Greenhouse-Geisser corrections were applied when a violation of sphericity in Mauchly’s tests was detected. Possible neurophysiological correlations were assessed using Pearson’s correlation test. The level of significance was set at *p* < 0.05 and Tukey’s test was used in post-hoc analysis to correct for multiple comparisons. Statistical analysis was performed using STATISTICA (TIBCO software inc., Palo Alto, CA, USA).

## 3. Results

The experimental procedures were well tolerated by all subjects and none reported adverse effects or any skin sensation during or after TUS. In all subjects, the electrical stimulation of the right supraorbital nerve evoked a reliable R2 response in the ipsilateral and contralateral orbicularis oculi muscle, while the intensity used was insufficient to induce a reliable R1 response.

As expected, the rmANOVA conducted on the unconditioned R2 only showed a significant effect of the factor ‘SIDE’ (F_1,15_= 19.81, *p* < 0.001), indicating that the R2 size was higher in the muscle ipsilateral (right) than contralateral to the stimulation. The main factors ‘CONDITION’ (F_3,45_ = 1.80, *p* = 0.16) and ‘TIME-POINT’ (F_2,30_ = 2.71, *p* = 0.09), as well as the ‘SIDE’ × ‘CONDITION’ (F_3,45_ = 0.56, *p* = 0.64), ‘SIDE’ × ‘TIME-POINT’ (F_2,30_ = 2.01, *p* = 0.15), ‘CONDITION’ × ‘TIME-POINT’ (F_6,90_ = 1.70, *p* = 0.13) and ‘SIDE’ × ‘CONDITION’ × ‘TIME-POINT’ (F_6,90_ = 1.69, *p* = 0.13) interactions were non-significant.

**Figure 4:**
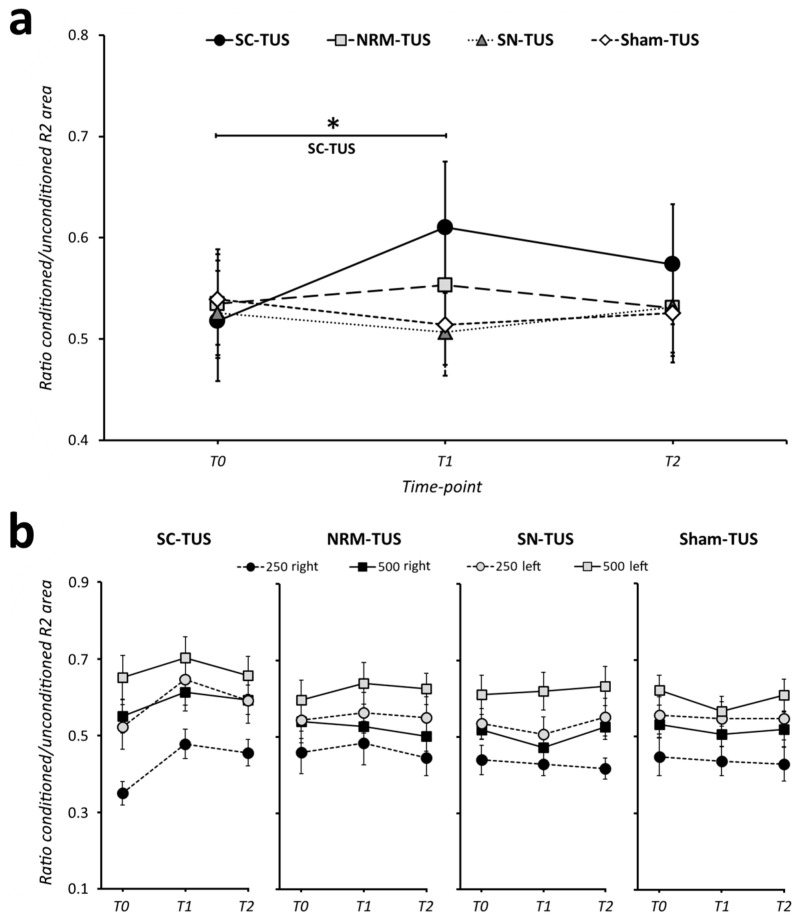
Effects of transcranial ultrasound stimulation on blink reflex excitability. (**a**) Transcranial ultrasound stimulation (TUS) delivered for 180 s on the superior colliculus (SC) increased the conditioned R2 area (i.e., lower R2 inhibition) at 3 min (T1) post-stimulation. In contrast, nucleus raphe magnus (NRM), substantia nigra (SN), and sham-TUS had no significant effects on blink reflex excitability. Since statistical analysis demonstrated that the effect was independent of the interstimulus interval (ISI) tested (250 or 500 ms) and R2 recording side (right or left), the panel shows averaged values. Error bars reflect the standard error of the mean. The asterisk denotes significant differences at post-hoc analysis. (**b**) Effects of SC, NRM, SN, and sham-TUS on the conditioned R2 area at the two ISI tested in the right and left side. Error bars reflect the standard error of the mean. T0: before TUS; T1: 3 min after TUS; T2: 30 min after TUS.

The rmANOVA conducted on the conditioned R2 highlighted a significant ‘CONDITION’ × ‘TIME-POINT’ interaction (F_6,90_ = 2.92, *p* = 0.01), indicating a time-dependent modulation of the R2 component depending on the stimulation condition. Post-hoc analysis demonstrated a higher conditioned R2 area at T1 than at T0 (*p* < 0.01) in the superior colliculus-TUS session, while no difference was present between T0 and T2 (*p* = 0.41) (Figure 4; Figure S1 shows individual data for superior colliculus-TUS). In contrast, in all other stimulation conditions the conditioned R2 area did not change after TUS (nucleus raphe magnus-TUS: T0 vs. T1 *p* = 0.99, T0 vs. T2 *p* = 1.0; substantia nigra-TUS: T0 vs. T1 *p* = 0.99, T0 vs. T2 *p* = 1.0; sham-TUS: T0 vs. T1 *p* = 0.99, T0 vs. T2 *p* = 0.99). As expected, the analysis also demonstrated a significant effect of the main factors, ‘SIDE’ (F_1,15_ = 11.92, *p* < 0.01) and ‘ISI’ (F_1,15_ = 42.51, *p* < 0.001), suggesting that the area of the conditioned R2 component was lower in the muscle ipsilateral (right) than contralateral to the stimulation, and lower at 250 ms than 500 ms ISI in all stimulation conditions and time-points considered. Finally, the rmANOVA did not show any effect of ‘CONDITION’ or ‘TIME-POINT’ or other interactions between factors (*p* always > 0.05, Table 1). In summary, the results suggest an increased conditioned R2 area immediately after superior colliculus-TUS, reflecting brainstem circuit excitability changes.

Finally, we tested whether the effects of superior colliculus-TUS (ratio conditioned R2 T1/T0, averaged across side and ISI) depended on the baseline level of blink reflex excitability in the same session (conditioned R2 at T0). The correlation analysis demonstrated no significant relationship between these measures (*r* = −0.36, *p* = 0.17).

## 4. Discussion

In the present study, we demonstrated that TUS targeting the superior colliculus, but not other brainstem areas, increased the conditioned R2 component area. This effect was present immediately after TUS, but not at a later time. Results were independent of the recording side (right or left) and the ISI tested (250 or 500 ms). These data suggest that superior colliculus-TUS modulates the excitability of the trigeminal blink reflex circuit in humans.

The double-blind, crossover, randomized, and sham-controlled experimental design allowed us to exclude that our results were biased by confounding factors. First, we carefully followed international standard procedures and we used well-known neuroanatomical landmarks to identify and target the various brainstem nuclei before applying TUS [28,29]. Our acoustic model demonstrates that despite the pressure wave being attenuated by the temporal bone, the targeted brainstem areas still received a proportion of the original ultrasound beam. Both participants and researchers who collected and analyzed the neurophysiological data were blinded to the stimulation conditions. The four experimental sessions were conducted at least 24 h apart, thus excluding possible carryover effects of TUS. In addition, sham-TUS did not induce any relevant effect on blink reflex recovery cycle, thus excluding the contribution of unspecific attentive or cognitive factors to blink reflex recovery cycle changes. In the latter regard, the area of unconditioned R2 was similar between the different sessions and time-points, possibly indicating no influence of unspecific arousal changes [36,37]. Finally, brainstem excitability at baseline was comparable between the different stimulation conditions, as shown by the similar unconditioned and conditioned R2 values before TUS in the various experimental sessions. We conclude that our results are essentially due to the putative physiological effects of TUS on the excitability of brainstem circuits controlling the blink reflex.

The main result of the study was the increased conditioned R2 area (both at 250 and 500 ms) immediately after TUS delivered on the superior colliculus, thus indicating early and short-lasting brainstem excitability changes. According to previous experimental studies on animals, the superior colliculus is thought to have a prominent role in modulating brainstem excitability [21,22,38]. The superior colliculus is a small, bilateral, and symmetrical nucleus in the tegmental midbrain area composed of several neuronal layers with intermediate and deep layers [22,39,40,41]. It receives inhibitory input via GABA projections from the substantia nigra and provides an excitatory output to the nucleus raphe magnus, which in turn inhibits brainstem excitability through 5 HT neurotransmission [21,22]. Accordingly, both direct (by stimulation of intermediate-to-deeper layers) and indirect (through inhibition of the substantia nigra) excitation of the superior colliculus caused a reduction in brainstem excitability in animals [21,22,39].

**Figure 5:**
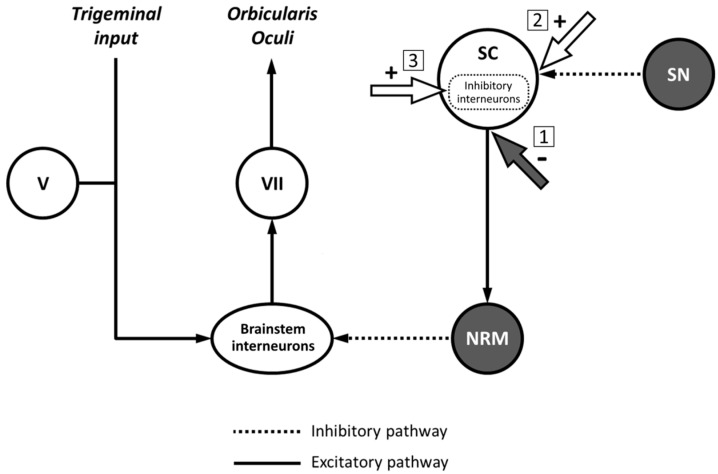
Possible mechanisms of action of transcranial ultrasound stimulation on the blink reflex circuit. Schematic representation of nuclei and circuits controlling blink reflex excitability (adapted from [21]) and possible mechanisms to explain the finding of reduced blink reflex inhibition after transcranial ultrasound stimulation (TUS). When delivered on the superior colliculus (SC), TUS may directly inhibit fibers originating from the SC and projecting to the nucleus raphe magnus (NRM) (1). Another possibility is that TUS exerts an excitatory effect at the level of GABAergic inhibitory synapses between substantia nigra (SN) projections and SC neurons (2). A third possible mechanism implies the facilitation of the activity of inhibitory interneurons located within the SC, thus reducing the output of the nucleus itself (3).

Several hypotheses may explain the increased conditioned R2 area after TUS delivered on the superior colliculus. The first hypothesis is that TUS exerts a direct inhibitory effect on deeper layers and output fibres of the superior colliculus projecting to the nucleus raphe magnus. Accordingly, nucleus raphe magnus activity would be reduced, resulting in a less inhibited blink reflex (Figure 5). However, evidence from animal studies indicates that ultrasound-induced currents determine excitatory rather than inhibitory effects, as demonstrated by increased neuronal firing [1,2,3,42,43] and enhanced neurotrophic factor levels [2,44,45]. Similar evidence in humans suggests that TUS has excitatory effects when it is delivered with the parameters we used (including frequency and stimulation duration) [6,7,10]. Hence, the hypothesis that brainstem excitability changes after TUS delivered on the superior colliculus are due to inhibitory effects on the deeper layers and output fibres of this nucleus is unlikely. A second hypothesis is that the increased R2 area after superior colliculus-TUS is due to excitatory effects of the stimulation on GABAergic inhibitory synapses between substantia nigra and superior colliculus neurons (Figure 4) [21,22]. In this case, TUS would indirectly inhibit superior colliculus output, thus producing nucleus raphe magnus activity depression and reduced inhibition of the blink reflex. However, we found no significant change in blink reflex excitability after substantia nigra-TUS. Therefore, we also exclude the hypothesis that TUS produces its effects through excitation of inhibitory projections from the substantia nigra to the superior colliculus. The hypothesis we favour is that TUS exerts excitatory activity on inhibitory interneurons within the superior colliculus. There is evidence that GABAergic interneurons within the superior colliculus downregulate the activity of the nucleus itself [40,46,47,48,49]. Also, a recent study demonstrated that TUS increases GABAergic inhibition at the cortical level [9]. Accordingly, TUS may enhance the activity of superior colliculus GABAergic interneurons and, in turn, produce an overall reduction in superior colliculus output to the nucleus raphe magnus, thus leading to an increased R2 area (Figure 4). We cannot exclude, however, that other inhibitory interneurons in the superior colliculus may also contribute to TUS effects. Since superior colliculus-TUS effects were only present early after stimulation and did not persist later in time, we speculate that TUS only produces short-lasting modifications in the excitability of these neurons rather than stable long-term synaptic plasticity mechanisms in blink reflex circuits [35,50]. These data are overall in line with the results of animal studies indicating transient effects of experimental manipulations on brainstem function, lasting from milliseconds up to two hours according to the methodology adopted [21,22,39].

The lack of substantia nigra and nucleus raphe magnus-TUS effects deserves a separate comment. Our results showed that the conditioned R2 area did not change before or after substantia nigra and nucleus raphe magnus stimulation, indicating comparable brainstem excitability before and after stimulation. We exclude that the lack of effect was due to a possible weak role of substantia nigra and nucleus raphe magnus in influencing the trigeminal blink reflex since several studies have demonstrated that they are both key structures in brainstem circuits [21,22]. Because of the relatively low focality of TUS, it is possible that substantia nigra or nucleus raphe magnus insonation had variable effects due to the coactivation of their facilitatory and inhibitory connections with multiple brain structures, resulting in no significant change in the trigeminal blink reflex excitability. Considering the results provided by our modelling, however, we favor the hypothesis that different brainstem areas have a variable sensitivity to the mechanical energy of the ultrasound beam. While relatively low pressure suffices to modulate superior colliculus interneurons, a higher energy is possibly needed to modify the activity of substantia nigra and nucleus raphe magnus. Specific biomechanical and bioelectrical properties of superior colliculus interneurons would make them particularly sensitive to TUS.

This study has some limitations. First, TUS has a relatively low spatial resolution [6,51], and thus we cannot exclude that regions outside of the targeted nuclei at brainstem level, where various structures and projections are tightly packed, were also insonated. Moreover, we have not performed anatomical or functional imaging investigations to better target the nuclei of interest [28]. Also, a recent study in animals found that ultrasonic neuromodulation possibly modifies cortical activity through indirect auditory mechanisms [52]. However, we believe this is unlikely to explain our results since the transducer position was similar in the different stimulation conditions, but the R2 changes occurred only during the superior colliculus-TUS. Finally, the superior colliculus is known to modulate not only eyelid blink but also saccadic eye movements [38]. However, monitoring eye movements was beyond the purpose of our study. Importantly, there is currently no standardized TUS protocol in humans, and different stimulation parameters (e.g., ultrasound power, duration, frequency, and stimulation pattern) may have variable results [6,7]. Therefore, our results are limited to the TUS parameters we used. In this regard, we cannot exclude that increasing the stimulation duration over 180 s determines a stronger superior colliculus modulation or effective changes in substantia nigra and nucleus raphe magnus activity. Future research may focus on these specific issues.

## 5. Conclusions

This is the first study investigating the effects of TUS on brainstem circuits in humans. We demonstrated that the conditioned R2 area increases early after the application of TUS on the superior colliculus suggesting a lack of brainstem excitability inhibition. The facilitatory effect of TUS on brainstem excitability may be explained by an excitatory action on GABAergic interneurons within the superior colliculus, while alternative hypotheses seem less likely. Overall, TUS is a safe, easy-to-use, and well-tolerated technique. A recent systematic review highlighted both excitatory and suppressive modulatory effects of TUS depending on the stimulation parameters and targeted areas [7]. Future studies assessing TUS effects on different deep and cortical neuronal circuits may provide further details on its mechanism of action. Again, the behavioral counterpart of brainstem TUS, e.g., changes in eye movements particularly due to the stimulation of superior colliculus, deserve further investigations [53]. Finally, TUS may be applied in pathological conditions characterized by altered brainstem function [25] to verify whether modulating abnormal brainstem excitability improves specific symptoms.

## Figures and Tables

**Table 1:** Results of rmANOVA conducted on unconditioned R2 values.

Factors and Interactions	F	d,f	*p*
‘SIDE’	11.92	1,15	**<0.01**
‘CONDITION’	1.02	3,45	0.39
‘TIME-POINT’	2.09	2,30	0.14
‘ISI’	42.51	1,15	**<0.001**
‘SIDE’ × ‘CONDITION’	0.13	3,45	0.94
‘SIDE’ × ‘TIME-POINT’	0.16	2,30	0.86
‘CONDITION’ × ‘TIME-POINT’	2.92	6,90	**0.01**
‘SIDE’ × ‘ISI’	2.40	1,15	0.14
‘CONDITION’ × ‘ISI’	1.55	3,45	0.21
‘TIME-POINT’ × ‘ISI’	1.03	2,30	0.37
‘SIDE’ × ‘CONDITION’ × ‘TIME-POINT’	0.70	6,90	0.65
‘SIDE’ × ‘CONDITION’ × ‘ISI’	2.53	3,45	0.07
‘SIDE’ × ‘TIME-POINT’ × ‘ISI’	1.16	2,30	0.33
‘CONDITION’ × ‘TIME-POINT’ × ‘ISI’	0.69	6,90	0.66
‘SIDE’ × ‘CONDITION’ × ‘TIME-POINT’ × ‘ISI’	1.33	6,90	0.25

## Data Availability

The datasets analyzed during the current study are available from the corresponding author upon reasonable request.

## Supplementary Materials

The following are available online at https://www.mdpi.com/article/10.3390/brainsci11050645/s1, Figure S1: Effects of superior colliculus-transcranial ultrasound stimulation (SC-TUS) on blink reflex excitability at the individual subject level.
