# Supplementary material for: Effects of Transcranial Ultrasound Stimulation on Trigeminal Blink Reflex Excitability
Source: Brain Sci. 2021 May 15;11(5):645. doi: 10.3390/brainsci11050645 (PMC8156436; doi:10.3390/brainsci11050645)
Supplement: Supplementary file 1 [file brainsci-11-00645-s001.zip › brainsci-1190022-supplementary.pdf]

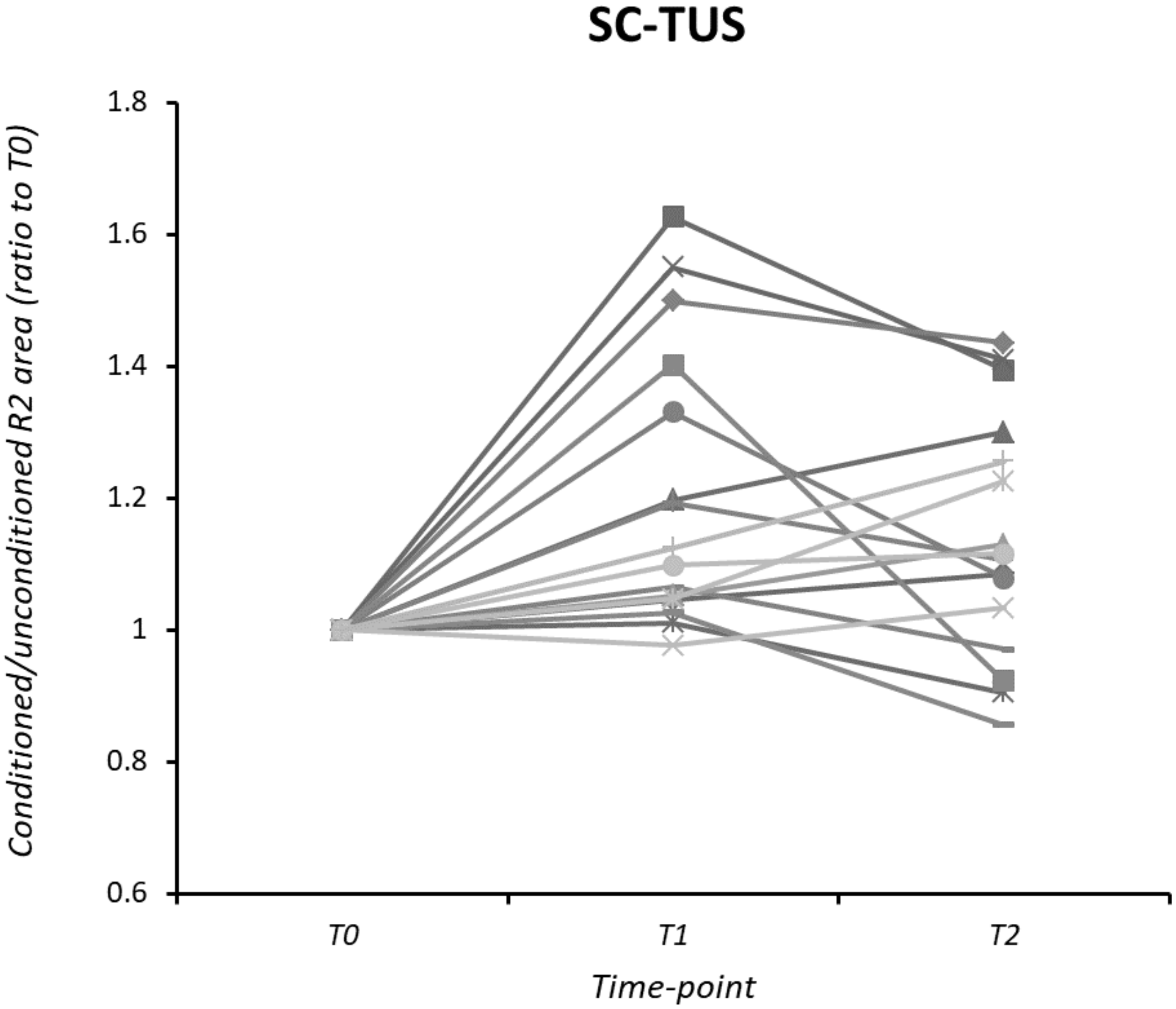

Figure S1. Effects of superior colliculus-transcranial ultrasound stimulation (SC-TUS) on blink reflex excitability at the individual subject level. Conditioned/unconditioned R2 area at T1 and T2 are normalized to T0. Since statistical analysis demonstrated that the effect was independent of the interstimulus interval (ISI) tested (250 or 500 ms) and R2 recording side (right or left), data reflect averaged values.
